# The Air of the Schoolroom

**Published:** 1895-08-17

**Authors:** 


### The Air of the Schoolroom.

The researches which have resulted in showing that school influence is a very potent factor in the dissemination of many diseases, will be far from bearing full fruition unless they have the effect of leading school managers to provide far better means of ventilating the buildings under their care than are at all common at the present time. Compulsory education throws on those who are entrusted with its supervision many responsibilities which pressed upon them in far less degree in days when people were free to take their education or to leave it, as they liked; and one of these responsibilities is, that in any case the children should not be injured by going to school. However great the benefits of elementary education may be—and we should be the last to throw doubts upon them—it can hardly be asserted that they are obtained without considerable risk, seeing how markedly the intercommunication of children at school becomes a means of diffusing ring worm, scarlet fever, diphtheria, measles, and other diseases. In view of this one of the first and most important points in school management should be the provision of a sufficiency of pure air, and of means for its frequent renewal by ventilation. That neither of these is at present provided is abundantly obvious to all who have to do with schools. We are free to admit that in many ways the surroundings of the children within the schools are better than in the homes from which many of them come, and that some of the newer schools are admirably designed for educational purposes. But on the other hand it must be remembered that the risks run by breathing air which has been breathed by others, and absorbing the dust, the effluvia, and the infection introduced upon the bodies and clothes of others, is increased in proportion to the numbers brought together, and the varieties of homes from which they come; and that, for the sake of protection from these risks, a far greater purity of air is requisite where many are gathered together, than in homes where the same small group of individuals habitually meet only each other.

Under these circumatances we would draw attention to the rules issued this year by the Education Department in regard to the ventilation of new schools—rules which we may presume are intended to enforce a standard superior to that which is at present commonly found in schools. "Apart from open windows and doors, there should be provision for copious inlet of fresh air," and they go on to say, "Inlets should provide a minimum of two and a-half square inches per child, and outlets a minimum of two inches." Now, to understand the meaning of this, let us compare it with the area considered desirable in the case of ordinary living-rooms. Whitelegge says, "The size of openings required for the efficient ventilation of an occupied room under average conditions is about 48 square inches *per head*, namely, 24 as an inlet and 24 as an outlet," and the model bye-laws issued by the Local Government Board state that "every habitable room must either have a fireplace and chimney or a special ventilating aperture or air-shaft with an unobstructed sectional area of at least 100 square inches." That is, even a single bed-room must have 100 square inches of ventilation independently of the window. Compare that with the 2½ square inches, at last demanded by the Education Department for each child in a school-room!

If, as authorities say, 3,000 cubic feet of fresh air per person per hour are required to keep the air of a room sweet, what are we to think of the rate at which it must flow through this tiny opening? Even if we give each child an hourly supply of only 2,000 cubic feet of air it would have to flow in at about twenty miles an hour, a speed which would be absolutely unbearable. As a fact it becomes necessary to trust to the windows, for the special appliances for ventilating schools rarely give the children sufficient air, and the rooms would become intolerable were it not that when the classes are changed the windows are opened and the foul air is swept out.

This complete flushing, however, occurs only at considerable intervals. The Education Department asks that the rooms should be flushed with fresh air every two hours, but that is a totally inefficient means of keeping them sweet. "A thousand cubic feet of air is a sufficient supply for one person for twenty minutes only," and yet we are only asked to flush the rooms once in two hours, although even in some of our newest London Board Schools, probably the best in the country, each child has only 156 cubic feet of air space, and the Education Department demands far less. Taking these facts into consideration on the one hand, and on the other bearing in mind the effect of "school influence" in the production of diphtheria and other diseases, as long ago pointed out by Dr. Thorne Thorne, and lately demonstrated in regard to London by Dr. Shirley Murphy, we cannot refrain from expressing the opinion that one of the first duties of our school managers to a community which is now forced to send its children to school is the provision of a far more complete system of ventilation than is suggested even by the most recent utterances of the Education Department.

